# Effect of an ethanol extract of *Descurainia sophia* seeds on Phase I and II drug metabolizing enzymes and P-glycoprotein activity *in vitro*

**DOI:** 10.1186/s12906-015-0965-0

**Published:** 2015-12-18

**Authors:** Jin-Mu Yi, Young Ah Kim, You Jin Lee, Ok-Sun Bang, No Soo Kim

**Affiliations:** KM-Convergence Research Division, Korea Institute of Oriental Medicine, 1672 Yuseong-daero, Yuseong-gu, Daejeon, 34054 Republic of Korea; Department of Korean Medicine, Life Science and Technology, Korea University of Science and Technology, Daejeon, Republic of Korea

**Keywords:** *Descurainia sophia*, Drug metabolizing enzyme, Herb-drug interaction, Inhibitory effect

## Abstract

**Background:**

*Descurainia sophia* seeds have a variety of pharmacological functions and been widely used in traditional folk medicine. However, their effects on human drug metabolizing enzyme (DME) activities have not been elucidated. The present study investigated the inhibitory effects of an ethanol extract of *D. sophia* seeds (EEDS) on human Phase I/II (DMEs) and P-glycoprotein (p-gp) *in vitro*.

**Methods:**

The enzyme activities of human Phase I (cytochrome P450s, CYPs), Phase II (uridine diphosphate glucuronosyltransferases, UGTs) DMEs, and the drug transporter P-gp were determined in the presence of various concentrations of EEDS using commercially available luminogenic assay systems. The mode of enzyme inhibition and the inhibitory constant (K_i_) value of EEDS were graphically determined with Lineweaver-Burk double reciprocal plots and secondary plots, respectively.

**Results:**

The enzyme activity assays showed that EEDS moderately inhibited the CYP1A2, CYP2C9, and CYP2C19 isoforms with half maximal inhibitory concentrations (IC_50_) of 47.3, 25.8, and 38.7 μg/mL, respectively. Graphical analyses with Lineweaver-Burk double reciprocal plots and secondary plots indicated that EEDS competitively inhibited CYP2C9 with a K_i_ value of 19.8 μg/mL; however, it inhibited CYP2C9 and CYP2C19 in a mixed mode with K_i_ values of 5.2, and 11.9 μg/mL, respectively. Other Phase I (CYP2C8, CYP2D6, and CYP3A4) and Phase II (UGT1A1 and UGT2B7) enzymes as well as P-gp were weakly or negligibly affected by EEDS with concentrations up to 500 μg/mL.

**Conclusions:**

EEDS is a selective inhibitor of CYP1A2, CYP2C9, and CYP2C19 with moderate enzymatic inhibition. Clinically, full consideration should be given to a potential toxic adverse effect from a herb-drug interaction when drugs that are particularly susceptible to CYP1A2, CYP2C9, or CYP2C19-mediated metabolism are taken together with EEDS. Characterization of metabolic profiles of specific herbal drugs could help consumers and medical specialists to use them safely as a complementary and alternative medicine.

## Background

In the past few decades, complementary and alternative medicine (CAM) has been combined with conventional medicine to treat diverse diseases. Approximately 38 % of American adults (83 million) are using some form of CAM for their healthcare, and their expenditures on CAM reached $34 billion in 2007 [1]. In Eastern Asia, the prevalence of CAM is much higher, and 76 % of Japanese [2] and 93 % of Chinese cancer patients have used CAM [3]. The proportion of population using CAM in the Republic of Korea was reported as 75 % [4]. In addition, herbal medicines are classified and regulated as formal drugs in these Eastern Asian countries [5].

Herbal medicines comprise an important part of CAM and are used worldwide. Consumers of herbal medicines consider them safe and sometimes forget the fact that herbal extracts contain many phytochemicals and that they can act as substrates, inhibitors, or inducers modulating human metabolic enzymes [6]. The increasing practice of co-administering herbal medicines with conventionally prescribed medications has raised considerably the risk of potential drug-herb interactions, especially in patients who already are on complex treatments involving narrow therapeutic index drugs [1, 7, 8]. Pharmacokinetic herb-drug interactions are one of the major risks caused by phytochemicals modulating the activities of enzymes that metabolize drugs, which subsequently affects their bioavailability [9]. For example, St. John's wort can decrease the steady state plasma concentrations of cyclosporine, midazolam, tacrolimus, amitriptyline, digoxin, indinavir, warfarin, phenprocoumon, and theophylline [10]. Ginkgo could enhance the hydroxylation of omeprazole by CYP2C19 in a genotype-dependent manner, and garlic could increase the EC_50_ of warfarin in wild-type vitamin K epoxide reductase complex subunit 1 (VKORC1) in patients [1]. The whole extract of kava, a well-known herbal anxiolytic, can inhibit human CYP1A2, CYP2C9, CYP2C19, CYP2D6, and CYP3A4 *in vitro* [11] and increase the area under the concentration-time curve (AUC) and the maximum achieved plasma concentration (C_max_) of co-administered kawain *in vivo* [12]. These studies show that determining the effects of individual herbal drugs on human drug metabolizing enzymes (DMEs) is important to predict possible interactions with drugs which can potentially be co-administered in clinics and to reduce the risk of adverse side effects from their concomitant use. Therefore, there is no doubt that chronic users of herbal medications, which are known to inhibit or induce potent human DME activities, should be aware of the potential adverse side effects from herb-drug interactions as well as be carefully advised by medical specialists before their use.

*Descurainia sophia* (L.) Webb ex Prantl (Flixweed), which belongs to the family Brassicaceae (Cruciferae), originates from Southern Europe and North Africa, and is widely distributed in Northeastern China [13]. The ethnopharmacological uses of *D. sophia* seeds are to relieve coughing and asthma, treat jaundice, reduce edema, promote urination, etc. [14]. A recent randomized clinical trial showed that the oral administration of *D. sophia* seeds relieved chronic functional constipation in children which was statistically significant [15]. Some research groups including us have identified various types of secondary metabolites from *D. sophia* seeds. They are cardiac glycosides, sinapoyl glycosides, flavonoids, lactones, lipids, coumarins, nor-lignans, lignans, and phytosterols [14, 16–21]. The biological activities of some of the isolated phytochemicals are anti-asthmatic, anti-tussive, expectorant [13], and anti-inflammatory as well as cytotoxic activities against human cancer cells [21]. Our previous genome-wide expression analysis showed that the solid fraction of the ethanol extract of *D. sophia* seeds regulated diverse genes which are closely associated with several biological functions like cell survival and death [22].

As evidence for the ethnopharmacological use of *D. sophia* seeds increases, the clinical applications and medicinal requirements of *D. sophia* seeds may increase gradually. However, no scientific study is currently available showing the effects of *D. sophia* seeds on the activities of human DMEs. The goal of the present study was to elucidate the inhibitory effect of *D. sophia* seeds on major human DMEs including Phase I (cytochrome P450s, CYPs) and Phase II (uridine diphosphate glucuronosyltransferases, UGTs) enzymes and a drug transporter (P-glycoprotein, P-gp).

## Methods

### Plant materials and extract preparation

The dried seeds of *D. sophia* were purchased from Kwangmyungdang Medicinal Herbs Co. (Ulsan, Republic of Korea). *D. sophia* seeds were collected in the Shandong Province, China in June 2010 which was written on the batch label. They were identified by Dr. Go Ya Choi at the K-herb Research Center, Korea Institute of Oriental Medicine, Daejeon, Republic of Korea. A voucher specimen (KIOM-CRC-5) was deposited at the KM-Convergence Research Division, Korea Institute of Oriental Medicine. Whole extract of the dried seeds of *D. sophia* was prepared by repeated maceration in 80 % (v/v) ethanol. The solid phase of the ethanol extract (EEDS) was further concentrated and dried. Finally, the EEDS was stored at 4 °C. The EEDS was dissolved in 100 % dimethylsulfoxide (DMSO, Sigma, St Louis, MO, USA) to make a stock solution and then stored at -80 °C until used. A detailed procedure on preparing EEDS was described previously [22].

### Luminogenic DME inhibition assays *in vitro*

*In vitro* enzymatic inhibition assays of human Phase I (CYPs) and Phase II (UGTs) DME, and P-gp were done with commercially available luminogenic assay systems. The assay systems were purchased from Promega (Madison, WI, USA), and the microsomes containing the recombinant human CYPs were purchased from Life Technologies (Carlsbad, CA, USA). The microsomes containing human UGT and P-gp were supplied in the luminogenic assay systems (Promega). The details on the assay systems and the microsomes are presented in Table 1. The activity for each enzyme was determined in the presence and absence of serially diluted EEDS. A known specific enzyme inhibitor was included as a positive control to verify the validity of each assay system. They are also summarized in Table 1. The maximum tolerable concentration of the vehicle (DMSO) was adjusted to 0.25 % (v/v) for all enzyme activity assays except for the DMSO-sensitive CYP2C8 (0.05 %). The enzyme reaction was done according to the manufacturer’s instructions. The luminescent intensity, which is correlated with the enzyme activity, was quantified with a TriStar LB 941 multimode microplate reader (Berthold Technologies, Bad Wildbad, Germany). The modulated enzyme activity by EEDS was evaluated by comparing the changes in activity from the treatment with the solvent alone to those from the treatment with the EEDS. The apparent half-maximal inhibitory concentration (IC_50_) was determined with the SigmaPlot program (version 11.0, Systat Software, Inc, San Jose, CA, USA) with a 4-parameter logistic equation.

**Table 1:** Summary of assay systems for human Phase I/II DME and P-gp inhibition assays

Types	DMEs	Assay kits		Microsomes		Specific inhibitors		
		Suppliers	Cat #	Suppliers	Cat #	Chemicals	Suppliers	Codes
Ph I	CYP1A2	Promega	V8772	Invitrogen	P2792	α-naphthoflavone	Sigma-Aldrich	N5757
	CYP2C8	Promega	V8782	Invitrogen	PV6138	Montelukast	Cayman	10008318
	CYP2C9	Promega	V8792	Invitrogen	P2378	Sulfaphenazole	Sigma-Aldrich	S0758
	CYP2C19	Promega	V8882	Invitrogen	P2864	Ticlopidine	Sigma-Aldrich	T6654
	CYP2D6	Promega	V8892	Invitrogen	P2283	Quinidine	Sigma-Aldrich	Q3625
	CYP3A4	Promega	V8802	Invitrogen	P2858	Ketoconazole	Sigma-Aldrich	K1003
Ph II	UGT1A1	Promega	V2120	Microsome supplied in assay kit		Diclofenac	Sigma-Aldrich	D6899
	UGT2B7	Promega	V2130	Microsome supplied in assay kit		Diclofenac	Sigma-Aldrich	D6899
Drug transport	P-gp	Promega	V3601	Microsome supplied in assay kit		Na_3_VO_4_ supplied in assay kit		

### Determination of the modes of enzyme inhibition and the inhibitory constant (K_i_) values

The modes of enzyme inhibition and the K_i_ values were further determined for the enzymes with IC_50_ values less than 100 μg/mL. They were determined by incubating a series of EEDS concentrations and different concentrations of respective substrates for each enzyme using the luminescence-based method described earlier. The enzyme reaction conditions are summarized in Table 2. The mode of enzyme inhibition was graphically determined from the Lineweaver-Burk double reciprocal plots. The K_i_ value was calculated with the secondary plot representing the slope rate of the Lineweaver-Burk plot versus the EEDS concentrations (0, 12.5, 25, 50, and 100 μg/mL) [23].

**Table 2:** Summary of reaction conditions to determine modes of CYP enzyme inhibition and K_i_ values

Parameters	CYP1A2	CYP2C9	CYP2C19
Substrate (μM)	Luciferin-ME	Luciferin-H	Luciferin-H EGE
	(12.5 ~ 100)	(25 ~ 200)	(2.5 ~ 20)
Enzyme (pmol)	0.5	0.5	0.25
EEDS (μg/mL)	0-100	0-100	0-100
Incubation time (min)	10	30	20
Temperature (°C)	37	37	37

*Luciferin-ME* luciferin 6-methyl ether, *Luciferin-H* 6-deoxyluciferin, *Luciferin-H EGE* ethylene glycol ester of 6-deoxyluciferin

### Statistics

The data are presented as the means ± standard deviation (S.D.) for at least duplicate experiments. The means and S.D. were determined with the SigmaPlot program.

## Results

### Inhibitory effects of EEDS on human DME activities

The inhibitory effect of EEDS on human DME activities were evaluated with the *in vitro* luminescent assay described earlier. The calculated IC_50_ of the EEDS and specific inhibitors against each DME are presented in Table 3. The inhibitory potencies of the test drugs were classified as potent (IC_50_ ≤ 20 μg/mL for crude extract or IC_50_ ≤ 10 μM for a single compound), moderate (20 < IC_50_ < 100 μg/mL or 10 < IC_50_ < 50 μM), or weak (IC_50_ ≥ 100 μg/mL or IC_50_ ≥ 50 μM) [24]. According to this categorization, the EEDS exhibited a moderate inhibitory effect on the CYP1A2, CYP2C9, and CYP2C19 activities with an IC_50_ of 47.3, 25.8, and 38.7 μg/mL, respectively. However, CYP2C8 (IC_50_ > 100 μg/mL), CYP2D6 (IC_50_ = 137.5 μg/mL), and 3A4 (IC_50_ = 134.2 μg/mL) were weakly inhibited by EEDS. The effect of EEDS on UGT1A1 was very weak (IC_50_ = 335.1 μg/mL) and on UGT2B7 and P-gp negligible (IC_50_ > 500 μg/mL).

**Table 3:** IC_50_ of EEDS on human DMEs

Types	DMEs	Inhibitors	IC_50_^a^	
			EEDS (μg/mL)	Inhibitors (μM)
Ph I	CYP1A2	α-naphthoflavone	47.3 ± 7.0	0.22 ± 0.03
	CYP2C8	Montelukast	>100	0.31 ± 0.02
	CYP2C9	Sulfaphenazole	25.8 ± 1.9	0.13 ± 0.01
	CYP2C19	Ticlopidine	38.7 ± 2.5	0.39 ± 0.03
	CYP2D6	Quinidine	137.5 ± 0.3	0.08 ± 0.00
	CYP3A4	Ketoconazole	134.2 ± 3.5	0.25 ± 0.03
Ph II	UGT1A1	Diclofenac	335.1 ± 57.3	180.3 ± 29.6
	UGT2B7	Diclofenac	>500	179.4 ± 30.9
Drug transport	P-gp	Na_3_VO_4_	>500	3.40 ± 0.32

^a^Values are expressed as the means ± S.D. of at least duplicated experiments

### Determining the mode of enzyme inhibition and the K_i_ value

**Fig. 1:**
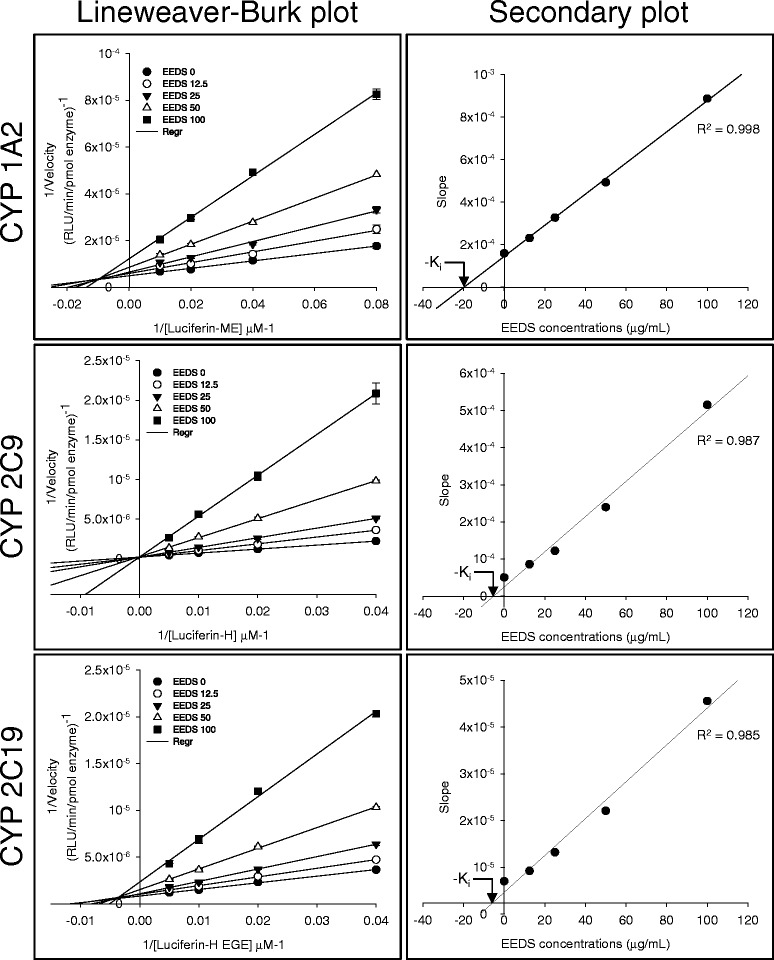
Left panel, primary Lineweaver-Burk plots for CYP1A2, CYP2C9, and CYP2C19. The modes of enzyme inhibition were determined by incubating an enzyme with a combination of different concentrations of EEDS and increasing the concentrations of specific substrates. Right panel, secondary plots for CYP1A2, CYP2C9, and CYP2C19. K_i_ value was calculated by the secondary plot representing the slope rate of the Lineweaver-Burk plot versus increasing the concentrations of EEDS. Minus K_i_ values (-K_i_) from the plot intersect on the x-axis

Next, we determined the K_i_ values and the mode of enzyme inhibition for the CYP1A2, CYP2C9, and CYP2C19 isoforms with an IC_50_ less than 100 μg/mL for the EEDS. From the results, the EEDS competitively inhibited CYP2C9 but showed mixed inhibition for CYP1A2 and CYP2C19 seen in the Lineweaver-Burk double reciprocal plots (Fig. 1). The calculated K_i_ values for CYP1A2, CYP2C9, and CYP2C19 were 19.8, 5.2, and 11.9 μg/mL, respectively (Fig. 1). Therefore, the most potent inhibition by the EEDS was for CYP2C9 followed by CYP2C19 and CYP1A2.

## Discussion

The effect of a potential drug candidate on human DMEs, such as the induction or inhibition of CYPs, can be a safety issue because it can change the metabolism of other co-administered xenobiotics or drugs unintentionally, and subsequently enhance or reduce their pharmacological or toxic profiles [25, 26]. *In vitro* enzymatic studies of human cytochrome P450 modulated by a specific compound are considered cost-effective in predicting the potential drug-drug interaction in clinical trials [27]. In this study, we evaluated the modulation of human DME and P-gp activities *in vitro* with ethanol extracts of *D. sophia* seeds. *D. sophia* seeds have been prescribed as a folk medicine worldwide to treat or relieve diverse diseases and symptoms, especially in eastern Asian countries. *D. sophia* seeds have been traditionally used as a component of herbal prescriptions. They have been taken as a water extract after decoction or a round pill just after grinding them. Therefore, taking into consideration the conventional intake methods, we used repeated maceration in 80 % ethanol to extract the active ingredients as much as possible.

In our previous study, we reported on the anticancer effects of EEDS against various human cancer cell lines and identified various bioactive constituents from EEDS [21, 22]. Fourteen compounds were isolated from the ethanol extract of *D. sophia* seeds by chromatographic separation methods. Among them, helveticoside showed the most potent cytotoxicity with an IC_50_ ranging from 0.034 to 0.596 μM depending on the human cancer cell line. Quercetin and syringaresinol exerted dose-dependent inhibitory effects on NO production in LPS-stimulated RAW264.7 cells with median effective doses (ED_50_) of 5.5 μM and 10.0 μM, respectively. Several research groups have reported on the biological activities of *D. sophia* seeds. Khodarahmi *et al*. evaluated the cytotoxic effects of volatile oil from *D. sophia* seeds on the MCF-7 and HeLa cell lines with an IC_50_ of 70 μg/mL and 180 μg/mL, respectively [28]. Gong *et al*. reported on the *in vivo* anti-asthmatic, anti-tussive, and expectorant activities of *D. sophia* seed oil supporting the ethnopharmacological use of *D. sophia* seeds to relieve respiratory diseases [13]. To the best of our knowledge, the present study is the first to report the effect of the whole extract of *D. sophia* seeds on human DMEs and P-gp. Biochemical luminogenic inhibition assays showed that human CYP1A2, CYP2C9, and CYP2C19 were moderately inhibited by EEDS based on their pharmacokinetic parameters (IC_50_ and K_i_). Other tested CYP isotypes including CYP2C8, CYP2D6, and CYP3A4, were weakly inhibited by EEDS. These results show that the bioavailability of drugs, which are metabolic substrates of CYP1A2, CYP2C9, or CYP2C19, can be affected when these drugs are concomitantly administered with EEDS, causing potential adverse effects by elevating the plasma levels of cytotoxic drugs, or leading to therapeutic failure by inhibiting the metabolic processing of inactive prodrugs. Among approved drugs, the substrates for CYP1A2 include amitriptyline (anti-depression) and erlotinib (anticancer). The substrates for CYP2C9 include ibuprofen (analgesic), warfarin (anticoagulant), and tamoxifen (anticancer). The substrates for CYP2C19 include diazepam, mephenytoin (anti-epileptic), methadone (analgesic) and bortezomib (anticancer) (DrugBank, www.drugbank.ca) [6]. Many nonsteroidal anti-inflammatory drugs (NSAID) are a substrate for CYP2C9. If *D. sophia* seeds as an herbal formulation and a NSAID like celecoxib, diclofenac, ibuprofen, or naproxen are co-administered to treat cold, the two drugs will compete with each other to bind with CYP2C9 [29, 30]. The decoction of *D. sophia* seeds has been used as an antipyretic for measles and smallpox [18], and to treat respiratory diseases in Korea (DongUiBoGam, a classic medical book on Koran medicine). If a patient is taking a drug for bronchitis, it is necessary to consider the drug interactions between the anti-inflammatory drugs of NSAIDs and the herbal formulation of *D. sophia* seeds. For more information of cytochrome P450 drug interactions, please refer to the following site (http://medicine.iupui.edu/clinpharm/ddis/).

Due to the fact that CYP3A4 metabolizes more than 50 % of the clinically prescribed drugs and represents the major metabolic enzyme in the human liver (40 %) and intestinal mucosa (80 %), CYP3A4 could contribute significantly to the metabolism of herbal drugs because most of the herbal drugs including EEDS are orally administered and metabolized primarily by the drug metabolizing enzymes in the liver and intestinal mucosa [6, 31, 32]. If EEDS is developed as a new anticancer herbal drug, EEDS could possibly be taken together with other conventional anticancer drugs. In our assay system, CYP3A4 was weakly inhibited by EEDS with an IC_50_ of 134 μg/mL. Therefore, EEDS may only slightly affect the bioavailability of anticancer drugs, such as dasatinib for chronic myelogenous leukemia and gefitinib for locally advanced or metastatic non-small cell lung cancer (DrugBank, www.drugbank.ca), whose primary administration route is oral. These drugs are metabolized and eliminated mainly by CYP3A4.

Graphical analysis with the Lineweaver-Bulk and secondary plots showed that the effect of EEDS on CYP2C9 is a competitive-type of inhibition. On the other hand, the effects of EEDS on CYP1A2 and CYP2C19 were best fitted to a mixed-type of inhibition. Competitive inhibition of EEDS implies that EEDS competes with a substrate (Luciferin-H used in this study) to bind to the active site of CYP2C9. Therefore, the inhibition of CYP2C9 by EEDS can be overcome by increasing the substrate concentration. The competitive inhibition of CYP is reversible which is most commonly observed in both *in vitro* and clinical settings [27]. In a non-competitive-type of inhibition, both the inhibitor and substrate can bind to the enzyme at the same time because they have separate binding sites on the enzyme, and therefore, the substrate or inhibitor does not affect the binding of the other to the enzyme. The mixed inhibition observed in CYP1A2 and CYP2C19 by EEDS is reversible and similar to noncompetitive inhibition except that the binding of the substrate (Luciferin-ME for CYP1A2 or Luciferin-H EGE for CYP2C19 in this study) affects the binding of the inhibitor (EEDS) vice and versa.

UGTs catalyze the glucuronidation reaction through which the glucuronic acid moiety of UDP-glucuronic acid is transferred to substrates or xenobiotics, which are eliminated from the human body [33]. Although UGTs are responsible for catalyzing approximately 35 % of the drugs metabolized by Phase II enzymes and also involved in the metabolism of several important endogenous substances such as bilirubin, steroid hormones, thyroid hormones, bile acid, and fat-soluble vitamins [34], the effect of herbal medicines on UGT activity has not been well characterized. Some phytochemicals isolated from Chinese herbal medicines are known to inhibit UGTs. For example, deoxyschizandrin and schisantherin A isolated from *Fructus schisandrae*, which has been used as a tonic in traditional Chinese medicine, potently inhibit the UGT1A3 isotype [35]. In the present study, we evaluated the inhibitory effect of EEDS on the UGT1A1 and UGT2B7 isotypes. From the results, EEDS was found to affect very weakly UGT1A1 (IC_50_ = 335.1 μg/mL) and UGT2B7 (IC_50_ > 500 μg/mL) in our biochemical assay system; thus, its effects on these two UST isotypes were negligible.

The P-gp protein is an ATP-dependent drug efflux transporter and also known as multidrug resistance 1 (MDR1) or ATB-binding cassette subfamily B member 1 (ABCB1). When the distribution of P-gp was determined with a monoclonal antibody raised against P-gp, the distribution of human P-gp in normal tissues was concentrated in cells found in the intestinal epithelium, liver, proximal tubule of the kidney, and capillary endothelium, where it pumps back toxic materials into the intestinal lumen, bile and urinary ducts, and capillaries, respectively [36–38]. Many studies reported that P-gp can interact with herbal decoctions or extracts and with phytochemicals isolated from medicinal herbs [36, 37, 39–41]. Administration of herbal medicines that affect P-gp activity can induce enhanced drug toxicity or improve intracellular drug transport by inhibiting the P-gp mediated drug efflux. In the present study, however, EEDS did not modulate the verapamil-stimulated ATPase activity of P-gp up to the maximum concentration of EEDS (500 μg/mL) tested in our biochemical assay system.

Some constituents of *D. sophia* seeds were evaluated for their effects on human DMEs. Quercetin, a flavonoid, is a potent competitive inhibitor of CYP1A2, CYP2C, CYP2C19 and CYP3A4 (K_i_ = 0.93, 1.67, 1.74 and 4.12 μM, respectively) [42], and inhibits human UGT2B17, a key steroid–metabolizing enzyme [43]. Additionally, Choi *et al* reported that enhanced doxorubicin absorption in the gastrointestinal tract is attributed to quercetin-induced inhibition of P-gp and that the reduced first-pass metabolism of doxorubicin is attributed to quercetin-induced inhibition of CYP3A in the small intestine [44]. Syringaresinol, another constituent of *D. sophia*, exhibited inhibitory effects on P-gp in adriamycin-resisitant human breast cancer cells, MCF-7/ADR [45].

## Conclusions

*In vitro* enzyme activity assays showed that EEDS modulates the activities of human Phase I/II DMEs and P-gp to varying degrees of potency indicating the potential risk of herb-drug interactions. CYP2C9 was competitively inhibited; however, CYP1A2 and CYP2C19 were inhibited in a mixed manner by EEDS with K_i_ values of 19.8, 5.2, and 11.9 μg/mL, respectively. CYP2C8, CYP2D6, CYP3A4, UGT1A1, UGT2B7, and P-gp were not weakly or negligibly affected by EEDS. In the present study, however, the bioactive constituent(s) responsible for affecting the enzyme activities were not identified. Further studies will have to be done to identify the responsible phytochemical constituents and their enzyme modulating properties. In addition, we should keep in mind that various *in vivo* factors could influence *in vitro* to *in vivo* extrapolation.

## Abbreviations

ABCB1: ATB-binding cassette subfamily B member 1
AUC: area under the concentration-time curve
CAM: complementary and alternative medicine
C_max_: maximum achieved plasma concentration
CYP: cytochrome P450
DME: drug metabolizing enzyme
DMSO: dimethylsulfoxide
ED_50_: median effective doses
EEDS: ethanol extract of *D. sophia* seeds
IC_50_: half maximal inhibitory concentrations
K_i_: inhibitory constant
MDR1: multidrug resistance 1
P-gp: P-glycoprotein
UGT: uridine diphosphate glucuronosyltransferases
VKORC1: vitamin K epoxide reductase complex subunit 1
